# Regarding: “Comparison of a single session of tDCS on cerebellum vs. motor cortex in stroke patients: a randomized sham-controlled trial”

**DOI:** 10.1080/07853890.2024.2348052

**Published:** 2024-04-30

**Authors:** Akiyoshi Matsugi

**Affiliations:** Faculty of Rehabilitation, Shijonawate Gakuen University, Daitou City, Osaka, Japan

We perused with great interest the scholarly contribution authored by Qurat-ul-Ain et al. featured in the Annals of Medicine [1]. The investigators conducted a Randomized Controlled Trial (RCT) to examine the efficacy of transcranial Direct Current Stimulation (tDCS) applied to the cerebellum and motor cortex in patients suffering from ataxia following cerebrovascular accidents. Despite the positive findings suggesting efficacy, it is crucial to engage in a nuanced interpretation of the results and address certain significant issues that merit consideration in the integration of these findings into a Systematic Review.

First, the temporal constraints of the study render it unfeasible to capture all the reported outcomes within the designated timeframe. [T2] is assessed 60 min after the cessation of the intervention, yet the comprehensive evaluation of the reported Berg Balance Scale (BBS) [2], Timed Up and Go (TUG), Balance Evaluation Systems Test (BESTest) [3], Montreal Cognitive Assessment (MoCA) [4], Mini-Mental State Examination (MMSE) [5], Timed 25-Foot Walk (T25-FW), Six-Minute Walk Test (6MWT), and Johns Hopkins Fall Risk Assessment Tool (JHFRAT) requires more than 60 min. Additionally, the authors allude to a 30 ∼ 40-minutes break following the T1 assessment. This inherent design flaw, hindering measurement feasibility, markedly diminishes the study’s reliability.

Secondarily, numerous issues arise concerning the outcome measurements. Notably, cognitive evaluations such as MMSE and MoCA without explanation using parallel version were administered thrice over approximately 3 ∼ 4 h. As is conventionally acknowledged, neuropsychological tests are highly sensitive to repetition and are unsuitable for a single-session study design completed within a single day. Furthermore, the JHFRAT, encompassing Age, Fall history, Medications, Patient Care Equipment, Mobility, and Cognition components, exhibits minimal variability in a brief duration. Nevertheless, the authors reported a mean change alongside a substantial standard deviation. This raises the plausible inference that the measured outcomes are inappropriate for this study and may be potentially inaccurately measured.

Thirdly, some concerns pertain to data handling. Initially, the authors should provide the sample size, mean difference, and standard deviation of the original data for calculating the effect size of 0.18 and the declaration of the parameters used for calculation with G*power to ensure reproducibility. Next, immediate public disclosure of the acquired data is imperative to substantiate the veracity of the study results.

The fourth distinctive feature involves the omission of crucial details regarding the stroke patients enrolled, including the severity of paralysis, the etiological pathology (hemorrhagic or ischemic), and the precise location of the lesion. Further, more precise information about parameter of setting for tDCS such as location and orientation of electrode can be provided. The non-disclosure of these parameters significantly restricts the generalizability of the results’ interpretation, hindering their applicability in systematic reviews and other methodological contexts, thereby complicating the discernment of heterogeneity.

While acknowledging the commendable aim of this study, critical deficiencies are apparent in the study design, analytical methodology, and reporting content. The authors should be meticulous in disclosing and explaining their data to address these concerns. Doing so would allow the results of this study to contribute effectively to the next intervention study.

## References

[1] Ahmad, Zafran, Ilyas, Saad, Ishtiaq, Summaiya, et al. Comparison of a single session of tDCS on cerebellum vs. motor cortex in stroke patients: a randomized sham-controlled trial. Ann Med. 2023;55(2):2252439. doi:10.1080/07853890.2023.2252439 38100750 PMC10732189

[2] Pickenbrock HM, Diel A, Zapf A. A comparison between the static balance test and the berg balance scale: validity, reliability, and comparative resource use. Clin Rehabil. 2016;30(3):288–293. doi:10.1177/0269215515578297. 25802425

[3] Padgett PK, Jacobs JV, Kasser SL. Is the BESTest at its best? A suggested brief version based on interrater reliability, validity, internal consistency, and theoretical construct. Phys Ther. 2012;92(9):1197–1207. doi:10.2522/ptj.20120056. 22677295

[4] Julayanont P, Phillips N, Chertkow H, et al. Montreal cognitive assessment (MoCA): concept and clinical review. In: Larner A, editors. Cognitive screening instruments. London: Springer; 2013. doi:10.1007/978-1-4471-2452-8_6.

[5] Molloy DW, Standish TI. A guide to the standardized Mini-Mental state examination. Int Psychogeriatr. 1997;9(S1):87–94. doi:10.1017/s1041610297004754. 9447431

